# Myosin XIK of *Arabidopsis thaliana* Accumulates at the Root Hair Tip and Is Required for Fast Root Hair Growth

**DOI:** 10.1371/journal.pone.0076745

**Published:** 2013-10-07

**Authors:** Eunsook Park, Andreas Nebenführ

**Affiliations:** Department of Biochemistry and Cellular and Molecular Biology, University of Tennessee, Knoxville, Tennessee, United States of America; Iwate University, Japan

## Abstract

Myosin motor proteins are thought to carry out important functions in the establishment and maintenance of cell polarity by moving cellular components such as organelles, vesicles, or protein complexes along the actin cytoskeleton. In *Arabidopsis thaliana*, disruption of the myosin *XIK* gene leads to reduced elongation of the highly polar root hairs, suggesting that the encoded motor protein is involved in this cell growth. Detailed live-cell observations in this study revealed that *xik* root hairs elongated more slowly and stopped growth sooner than those in wild type. Overall cellular organization including the actin cytoskeleton appeared normal, but actin filament dynamics were reduced in the mutant. Accumulation of RabA4b-containing vesicles, on the other hand, was not significantly different from wild type. A functional YFP-XIK fusion protein that could complement the mutant phenotype accumulated at the tip of growing root hairs in an actin-dependent manner. The distribution of YFP-XIK at the tip, however, did not match that of the ER or several tip-enriched markers including CFP-RabA4b. We conclude that the myosin XIK is required for normal actin dynamics and plays a role in the subapical region of growing root hairs to facilitate optimal growth.

## Introduction

Myosin motor proteins couple the hydrolysis of ATP to the active movement of organelles or macromolecular assemblies along the actin cytoskeleton and therefore are responsible for cytoplasmic streaming in plant cells [1]. Cytoplasmic streaming is important for the distribution of cellular components within a cell and can be expected to play a major role in establishing and maintaining cell polarity. A classical example of this process is the delivery of organelles and secretory vesicles into the growing daughter cell of budding yeast, *Saccharomyces cerevisiae* [2]. An analogous process occurs in plant cells that expand at a single point on their surface, i.e. in tip-growing pollen tubes and root hairs.

Root hairs are long tubular outgrowths of root epidermal cells that functionally increase the root surface for water and solute uptake. Tip growth of root hairs is regulated by a complex self-organizing feedback mechanism that involves numerous signaling molecules such as calcium ions, reactive oxygen species, membrane lipids and small G-proteins [3-5]. This results in a dynamic actin cytoskeleton which directs the steady state accumulation of a large number of small vesicles in the apical dome where secretion occurs [6]. A subset of these vesicles, that likely function as secretory vesicles [7], can be labeled with the small GTPase YFP-RabA4b [8]. Since accumulation of these vesicles during growth depends on the actin cytoskeleton [8], it is likely that myosin motors may play a role in their movement to the root hair tip. This conjecture is supported by the recent finding that mutants in two class XI myosins were found to affect the length of root hairs [9,10], supporting a role for these motors in this process.

Class XI myosins have a similar domain organization as myosin V, which are known to move organelles in animals and fungi [11]. Thus, class XI myosins are generally believed to drive rapid organelle movements during cytoplasmic streaming [1,12]. The similarity of these two myosin classes was further supported when it could be shown that the myosin XI globular tail domain can take on the same conformation as that of myosin V and also acts as the organelle binding domain [13]. Fluorescently tagged tail constructs revealed various subcellular localizations [13-16] and could also act as dominant negative inhibitors of organelle movements [14,16,17]. Curiously, subcellular localization of these dominant negative constructs did not coincide with the organelles that were affected [14], suggesting that unusual mechanisms such as direct inhibition of motor activity [17] may be at work.

Knockout mutants of individual myosin XI genes in angiosperms have resulted in relatively mild phenotypes, presumably due to the large number of paralogs in flowering plants [18-20]. In rice, mutations in Os*myoxib* resulted in male sterility during short days, suggesting that this myosin is necessary for normal pollen development under these conditions [21]. In tobacco, RNAi was used to establish a role for myosin XI-2 in virus spreading [22] and stromule formation on plastids [23]. In maize, the *opaque1* mutation is a myosin XI gene leads to abnormal protein body formation in endosperm tissue [24]. In *Arabidopsis*, single mutants had mostly no detectable phenotype, except for *mya2* and *xik* which resulted in shorter root hairs [9,10]. Higher order mutants resulted in progressively more pronounced defects, with some triple mutants (e.g. *mya2 xib xik*) nearly abolishing root hair elongation and quadruple mutants leading to dwarfed plants that have an abnormal actin organization [25-27]. Thus, individual plant myosins likely perform overlapping functions but as a group are essential for normal development. This conclusion is further supported by results from the moss *Physcomitrella patens*, where RNAi suppression of the two major myosin XI genes prevented normal morphogenesis [28].

While the general role of myosin XI motors in plants has been clearly established, the precise cellular function of individual myosin isoforms is still unclear. The short root hair phenotype of *xik* and *mya2* mutants [9,10] offers the possibility to dissect these functions in detail. In the past, both mutations have been shown to result in reduced movements of Golgi stacks, peroxisomes, and mitochondria in root hairs [10], but the relationship of these movements to reduced root hair growth has not been explored. XIK protein was detected in subcellular fractions that corresponded to ER membranes from above-ground organs [29], but not in roots [30]. Consistent with this localization, ER movements were reduced in leaf petiole epidermal cells. In addition, ER as well as actin filament organization was altered in leaf cells, although this was only visible in multiple myosin knockouts [29]. Thus, it was proposed that myosin XIK is responsible for ER streaming and that these movements indirectly affect cytosol streaming and actin filament organization [29]. A similar indirect effect may be responsible for movements of Golgi stacks, peroxisomes and mitochondria, although it is not known how these movements influence root hair growth. Recently, results of cell fractionation analysis of the distribution of myosin XIK and localization of XIK-YFP fusion protein implied that XIK might have multiple cargoes, possibly secretory vesicles [30], however, it is still not clear how loss of XIK leads to short root hairs in *xik* mutants.

In this study, we characterized the root hair growth defect of myosin *xik* mutants in detail to identify possible XIK functions during tip growth. In particular, we tested a number of live-cell markers for various subcellular compartments in WT and *xik* root hairs, and analyzed the dynamic behavior of actin filaments and regulatory proteins. Complementation of the mutant with a tagged XIK protein revealed its intracellular localization.

## Materials and Methods

### Mutant lines and plant growth

Mutant alleles were isolated from the SALK collection [31] and Col-0 was used as wild type. *xik-1* (SALK_136682) and *xik-3* (SALK_018764) have been described previously [9,10]. New insertional *xik* alleles identified in this study were SALK_028822 (*xik-6*) and SALK_059031 (*xik-7*). Additional alleles were described elsewhere: *xik-2* (SALK_067972) was isolated by [9], *xik-4* (SALK_152496) was identified by [29], while another allele (WiscDsLox417C12, “*xik-5*”) was described in [10]. Positions of all insertions are marked in Figure S1A.

Plants were grown in a growth chamber at 22°C in 16 h light and at 20°C in 8 h dark with about 60% humidity. For root hair length measurements, seeds were germinated on square plates of 1/4 strength MS with 1% sucrose, pH adjusted to 5.7 and solidified with 0.5% phytagel (Sigma-Aldrich). Plates were incubated at 10° off vertical in a growth chamber with continuous light. 5 day-old seedlings were used for the measurements.

### *xik* complementation test

The construct for the complementation test was driven by the *XIK* native promoter, which extended 1244 bp upstream of the *XIK* start codon and included the first exon and intron of *XIK* (corresponding to nucleotides 6,938,624 -6,936,531 in TAIR10; http://www.arabidopsis.org/). The promoter was followed by YFP (yellow fluorescence protein), a short linker DNA encoding ELYGGPGGSGSA, and the full-length *XIK* coding region. This construct was introduced to the binary plasmid pPZP221 (Genbank U10491 [32]) and transformed into *xik-3* mutants using the floral dip method [33]. A full-length *XIK* cDNA (GenBank accession JN229265) was assembled by combining two EST clones, BE526400 [34] and AV546218 [35], which cover the entire length of the gene including 5’ UTR and 3’ UTR.

Root hair lengths were measured in images of 5-day-old seedlings that were captured under a stereomicroscope (Leica MZ16 FA, http://www.leica-microsystems.com) equipped with digital camera (Leica DFC420) under 7.1X magnification or 23X magnification. Images were acquired with Leica FW4000 software. More than 150 root hair lengths from 15 or more plants in each genotype were measured with ImageJ (http://rsb.info.nih.gov/ij/) and statistically analyzed in Prism 5 (http://www.graphpad.com/).

### Root hair growth rate measurements

To measure root hair growth rate, special devices as described in [36] were used with a modification to maintain plants healthy during observation. Briefly, 4 day old vertically grown seedlings were moved to a cover glass-bottom culture dish (5 cm diameter; Electron Microscopy Sciences) and covered with 2 mL of media containing 1/4 strength MS salt, 1% sucrose, and 0.7% type VII agarose (Sigma-Aldrich) at pH 5.7. Culture dish chambers were sealed and placed in the growth chamber for up to one day. The culture dish chambers were tilted 30° off vertical so that roots would grow along the cover glass. DIC images of root hairs were taken with an Orca ER camera (Hamamatsu) in 30 sec. intervals for 45 min. or 1 hour under a 100X objective with binning turned off to maximize spatial resolution. Time-lapse capture was repeated 5-7 times to observe the entire growth of a root hair from shortly after bulging to fully-grown. Length of root hairs was measured with the measurement plug-in in Openlab 5 (Improvision). Data were analyzed and graphed with Prism 5.0.

### Marker constructs and plant transformations

Constructs and their transgenic plants are listed in Table S1 online. Briefly, *YFP/mCherry-ROP2* was generated from amplified cDNA sequences based on the published sequence information [37]. *YFP/CFP-RHD4* was constructed from amplified cDNA sequences with published primer information [38]. Constructs were transformed into wild type and *xik-3* mutants by Agrobacterium-mediated transformation [33]. Stable transgenic plants were screened with appropriate antibiotics or herbicides and observed under a fluorescence microscope.

For *EXP7pro:YFP/mCherry*, the DNA fragment containing the *EXP7* promoter was obtained by PCR from genomic DNA with *EXP7pro-F* (5’-GCTAGCTTAGTTTATCTTTGGAAACGAAACGTAA-3’) and *EXP7pro-R* (5’-CCATGGTTCTAGACCTAGCCTCTTTTTCTTTATTCTT-3’) ( [39]). The PCR product was cloned in pGEM-Teasy vector (Promega) and sequenced, then moved to a binary vector, pFGC19 ( [40]), with XbaI (on primer) and EcoRI (from T-vector) restriction.

### Microscopy

Unless noted otherwise, all microscopic observations were carried out with a 63x (1.4 NA) plan-apo oil immersion objective on an Axiovert 200M microscope (Zeiss, http://www.zeiss.com) with filter sets for CFP, YFP, and mCherry observation (filter sets 52017 and 69308, Chroma, http://www.chroma.com). For most observations, seedlings were mounted in a cover-slip bottom culture dish under a thin (2-3 mm) phytagel blanket containing standard growth medium. Images were captured at regular intervals with a digital CCD camera (ORCA-ER, Hamamatsu, http://www.hamamatsu.com) controlled by Openlab software (Improvision). For quantitative image analysis, a dark background image was captured under identical exposure settings and subtracted from all fluorescence images to remove camera noise.

### Analysis of YFP-RabA4b accumulation

35 *Spro:YFP-RabA4b* in pCAMBIA, kindly provided by E. Nielsen (University of Michigan, Ann Arbor, MI) was transformed independently into Col-0 and *xik-3*. homozygous T3 seeds were grown for four days on vertical plates as described above before imaging. Time-lapse sequences of YFP and DIC were separated and analyzed with a custom macro in ImageJ (available upon request). To measure coefficients of variation of YFP fluorescence at the root hair tip, fluorescence intensities were measured in the apical dome (Figure S2B). Raw intensity values were bleach corrected by normalizing to a predicted first-order exponential decay whose parameters were obtained by linear regression of log-transformed data. To capture the variability of the fluorescence intensity, the coefficient of variation was calculated by dividing the standard deviation by the mean intensity. Statistical analyses and graphing of results were performed with Prism 5.0.

### Analysis of actin dynamics

An actin marker, *d35Spro:YFP-FABD2*, was transformed into both wild type and *xik-3* mutant, and stable transgenic plants were screened for reliable fluorescence expression. Time-lapse images of 15 growing root hairs were taken in 1 s intervals for 2 min. with microscope settings as described above. Images were exported to and analyzed in ImageJ [41]. Actin dynamics were quantified by determining the decay of image cross-correlation over time [28] with the following modifications. Since decay constants depend strongly on the size of the selection and the region of the cell analyzed (data not shown), we separated the time lapse sequence into 6 x 6 µm^2^ squares and performed the calculations on each of them. Two series of squares were created with 3 µm displacement to avoid possible problems with inadequate selection of squares. For each square, cross-correlation was calculated for increasing intervals between all possible image frame pairs with a custom macro in ImageJ (available on request). A single-phase exponential decay was fitted to the data in Prism 5 (Graphpad Software Inc.) according to equation 1.

$$y=(y_{0}-p)\cdot e^{-K\cdot x}+p$$ (1)

Where y_0_ is the calculated cross-correlation for interval length 0, p is the calculated plateau of the curve, and *K* is the decay constant. It is usually not possible to reliably fit a decay curve for all squares in a time-lapse sequence, mostly due to weak fluorescence signals. To avoid these problematic areas, we selected the 10 squares with the largest span (span = y_0_ -p) for further analysis since these corresponded to the regions of the cell where the strongest actin signal was obtained. The decay constants *K* from these 10 squares were plotted and their average was used to perform a Mann-Whitney test between WT and *xik-3* cells.

### Analysis of actin organization

Actin filaments were identified by filtering smoothed fluorescence images with a four-fold linear (i.e. star-shaped) kernel which effectively identifies linear features analogously to multidirectional non-maximum suppression [42]. The resulting image was binarized with the “Isodata” threshold and the remaining lines were reduced down to single pixels with the “Skeletonize” command of ImageJ. The resulting line segments were filtered for a minimum length of 1 µm with the “Analyze Particles” command and measured for length and position in the cell. Image stacks were processed with a custom macro in ImageJ (available upon request).

### Analysis of ROP2 recruitment at the plasma membrane

Stable transgenic lines of *EXP7pro:mCherry/YFP-ROP2 and d35Spro:YFP-ROP2* were isolated in both wild type and *xik-3* background. Individual root hairs were imaged every 5 s for 3 min. Images were exported to ImageJ and analyzed by a custom macro (available upon request) that tracked the tip during growth and identified the position of the plasma membrane in the central half of the root hair. Fluorescence intensity of the plasma membrane as well as of the cytosol 0.5 µm behind the plasma membrane (Figure S3) was measured and exported to Excel (Microsoft, http://www.microsoft.com) for quantitative analysis. Statistical tests were performed in Prism 5 (GraphPad, http://www.graphpad.com).

### YFP-XIK localization analysis

*XIKpro:YFP-XIK xik-3* plants were observed for their YFP expression in root hairs. Microscope settings were identical to the analysis of RabA4b accumulation except that longer exposure times were used. Sequential images were obtained under 63X with 2X binning for 1 min with 1 s intervals.

For drug treatment, 5-day-old seedlings grown as described above were transferred to the observation chambers. Inhibitors were diluted in liquid media and applied underneath the solid media blanket covering the root of a seedling in the observation chambers (100 nM of LatB from 1 mM stock in DMSO). Images were taken for 60 min in 30 sec intervals and processed as described above.

### Colocalization analysis

For colocalization tests, *d35Spro:CFP-RABA4B, d35Spro:CFP-RHD4*, *EXP7pro:mCherry-ROP2*, and *EXP7pro:mCherry* were transformed into homozygous *XIKpro:YFP-XIK xik-3* plants. T1 seedlings were screened on media with double selection and moved to a vertical plate without selection for additional growth for a day to allow normal growth of root hairs. Once seedlings started producing normally shaped root hairs, they were moved to an observation chamber and sequential dual color images were acquired for one to 30 minutes in 1 to 10 s intervals.

Distribution of signal intensities along the root hair were determined with a custom macro in ImageJ (available on request) by calculating the average signal intensity per row of pixels at different distances from the root hair tip. Maximal signals were normalized to 100% for each frame of the time lapse sequence and normalized signal intensities were averaged over all time points (n typically 60 or 180). At least seven cells were analyzed in this way and the position of the maximal signals averaged for each of the markers.

## Results

### Root hairs of *xik* mutants grow more slowly and stop growing sooner than root hairs of wild type

Four T-DNA insertion mutants of *XIK* (At5g20490) from the SALK collection [31] were isolated and their T-DNA insertion site confirmed by genomic PCR (Figure S1A). Among them, *xik-1* and *xik-3* were identified as knockout mutants by RT-PCR in agreement with reports by other groups [9,10], while two new insertion alleles in the promoter region (*xik-6* and *xik-7*; Figure S1A) still expressed the transcript (data not shown). The presence of a strong root hair phenotype in *xik-6* suggests potential differences in expression levels that should be investigated in the future. A full-length cDNA was assembled from two independent EST clones. Its sequence (GenBank accession number JN229265) corresponded to the major transcript identified by deep sequencing (HQ427882) [43] and included a different 5’ end from the previous annotations (NM_001161252 and NM_122056). In particular, the transcriptional start site was approximately 200 bp further upstream and a different splice acceptor site was utilized for the first intron when compared to NM_001161252. As a result, the encoded protein had a different N-terminus that included only a short extension upstream of a SH3-like domain. Thus, the gene structure of *XIK* appears to be similar to other plant myosin XI genes with a long first intron (831 bp) that follows immediately after the start codon.

**Figure 1:**
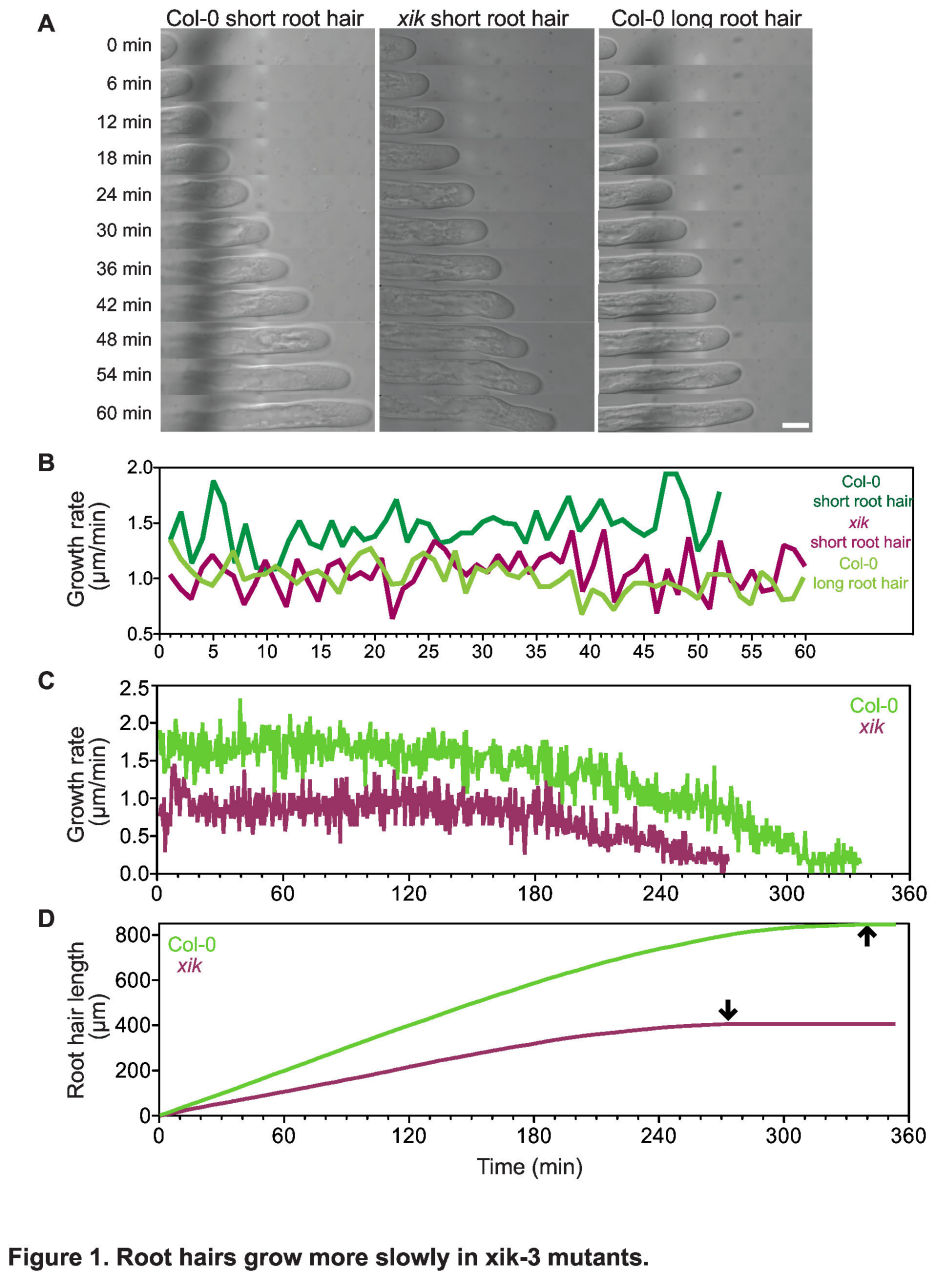
Root hairs grow more slowly in *xik* mutants. (a) Time series of DIC images of Col-0 and *xik*. Delay between images 6 min. Size bar indicates 15 μm. Young Col-0 root hairs (length < 200 µm) grew faster than those about to stop growing (length > 500 µm), compare first and third column. By contrast, young *xik* root hairs grew slowly and similarly to mature root hairs in Col-0 (compare second and third column). (b) Comparison of root hair growth rates over 60 min. Dark green line shows WT root hair growth rate of young root hairs, while light green indicates long WT root hairs. Magenta line shows the growth rate of a young root hair of *xik*. (c) Growth rates measured over 360 min reveal that root hairs in *xik* consistently grew more slowly than those of WT and eventually stopped growing earlier than in WT. Green line shows a root hair from a WT seedling, while magenta line indicates an individual root hair from a *xik* seedling. (d) Total root hair length over time for a *xik* root hair (magenta line) and a wild type root hair (green line). Arrows indicate approximate time point of cessation of growth.

Under our conditions, root hairs of *xik-3* (referred as *xik* below) usually grew to about 50-60% of root hair length in wild type, consistent with previous reports [9,10]. This phenotype could have resulted from two different mechanisms: mutant root hairs might have grown normally but stopped growing earlier than wild type, or they might have grown more slowly than wild type for the same duration. To distinguish between these possibilities, root hair growth rates were measured over time at high resolution on the microscope stage. This experiment was repeated four times per genotype and one representative result is shown in Figure 1. Under these conditions, *xik* mutants grew for 4-5 hours while wild type grew for up to about 6 hours (Figure 1D). Both wild type and mutants showed growth rate oscillations as reported previously [44]. As root hairs got older, their growth rate declined and eventually reached zero. However, comparison of growth rates of root hairs of similar age revealed that growth rates of *xik* were much lower than those of wild type (Figure 1B). Maximum root hair growth rate in *xik* under these conditions was 1.47 μm/min while wild type reached a maximum growth rate of 2.33 μm/min suggesting that root hairs in *xik* grew more slowly from the beginning (Figure 1C). Overall, average growth rates were 1.26 ±0.02 μm/min and 0.74 ±0.01 μm/min (SE) in wild type and *xik*, respectively (one way ANOVA, p< 0.001). In addition, *xik* stopped growing more than one hour earlier than wild type (Figure 1D, black arrows). Thus, *xik* mutants produced shorter root hairs than wild type because of a reduced growth rate as well as premature cessation of growth.

### RabA4b vesicles accumulate similarly in WT and xik root hairs

**Figure 2:**
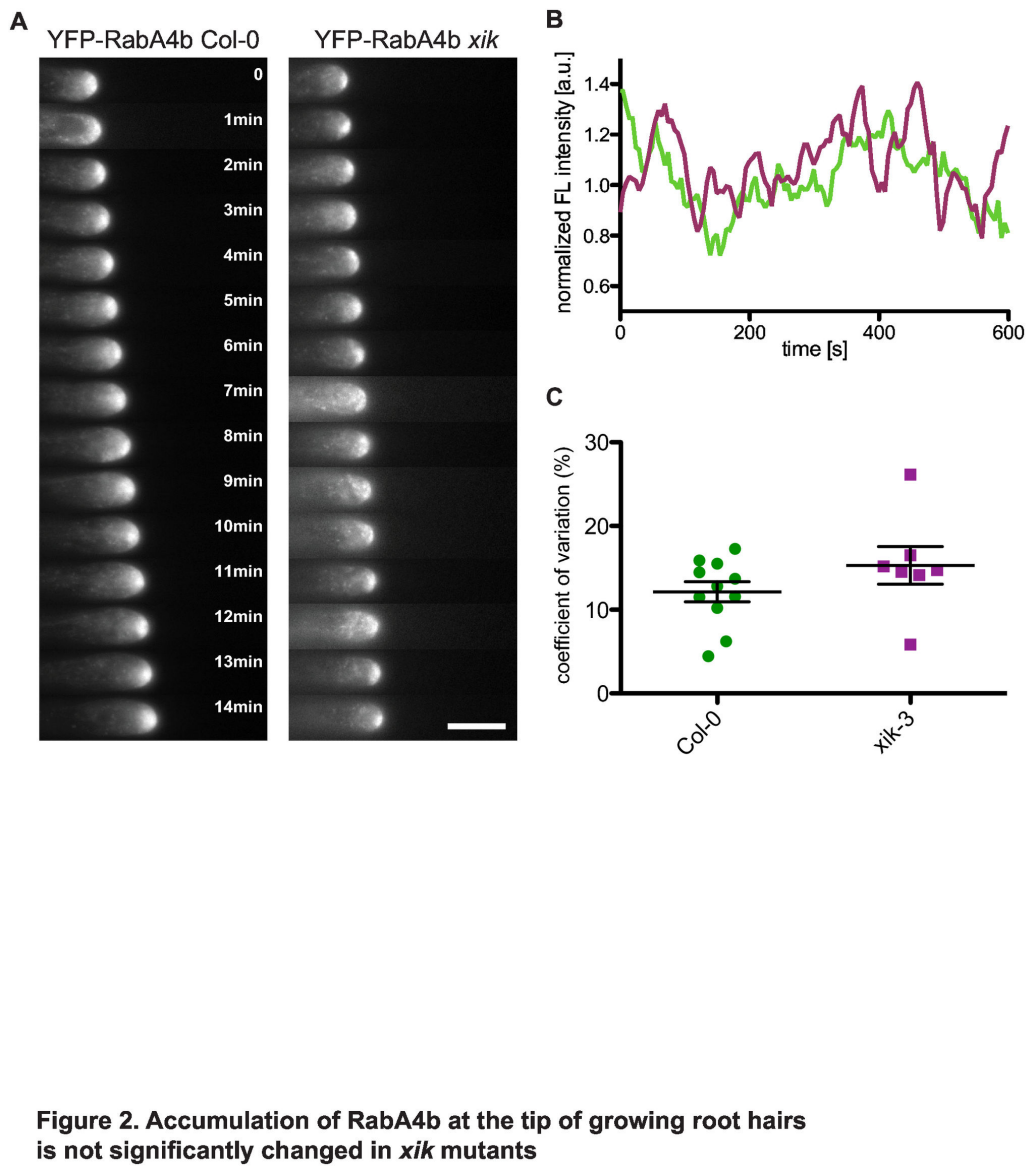
Accumulation of RabA4b at the tip of growing root hairs is not significantly affected in *xik* mutants. (a) Time series of YFP-RabA4b images of Col-0 and *xik*. Accumulation of YFP-RabA4b at the tip of growing root hairs fluctuated similarly in both Col-0 and in *xik*. Delay between images 1 min; also see Movie S1. Size bar indicates 10 μm. (b) Fluctuation of normalized fluorescence intensity of YFP-RabA4b during 10 min. Green line indicates data from Col-0 while magenta line indicates *xik*. Intensity fluctuations in *xik* were similar to those in Col-0. (c) Coefficients of variation of YFP-RabA4b accumulation in the tip. Every dot represents data from one root hair (n=15; Col-0, n=13; *xik*). Means of coefficients of variation were not significantly different in the two genotypes (t test, p > 0.05).

YFP-RabA4b associates with secretory vesicles that accumulate in the tip of growing root hairs and thus can be used as a marker for growth [7,8]. Since root hairs in *xik* mutants were growing more slowly and stopped growing earlier than in wild type, the effect of the *xik* mutation on YFP-RabA4b accumulation was observed. Root hairs from both wild type and *xik* mutants clearly displayed accumulation of YFP-RabA4b at the tip of growing root hairs (Figure 2A). A direct comparison of the level of YFP-RabA4b accumulation was not possible since high variation in expression levels even among root hairs of the same root (data not shown) prevented quantitative comparisons of wild type and mutant. To investigate the effect of the *xik* mutation on the accumulation of YFP-RabA4b in greater detail, YFP fluorescence at the tip of root hairs was monitored over time using an automated algorithm that tracked the root hair tip during growth (Figure S2). Interestingly, tip accumulation of YFP-RabA4b in both wild type and *xik* frequently displayed a stochastic loss and recovery at the tip of root hairs (Figure 2B and Movie S1 online). Coefficients of variation at the tip of *xik* root hairs were slightly higher but not significantly different from those of wild type (t-test: p= 0.1945, Figure 2C).

In addition, several other markers for membranes and the cytoskeleton were observed in *xik* mutants (listed in Table S1). In general, we did not observe clear differences in marker distribution between WT and *xik* root hairs. For example, the ER marker, ER-cb [40], showed the same distribution in WT and mutant root hairs (Figure S4A). Similarly, YFP-PH_FAPP1_, which binds to phosphatidylinositol 4-phosphate (PI4P) at the plasma membrane as well as to some intracellular vesicles [45], showed indistinguishable localization in the two genotypes (Figure S4B).

### Actin dynamics are reduced in *xik* root hairs

**Figure 3:**
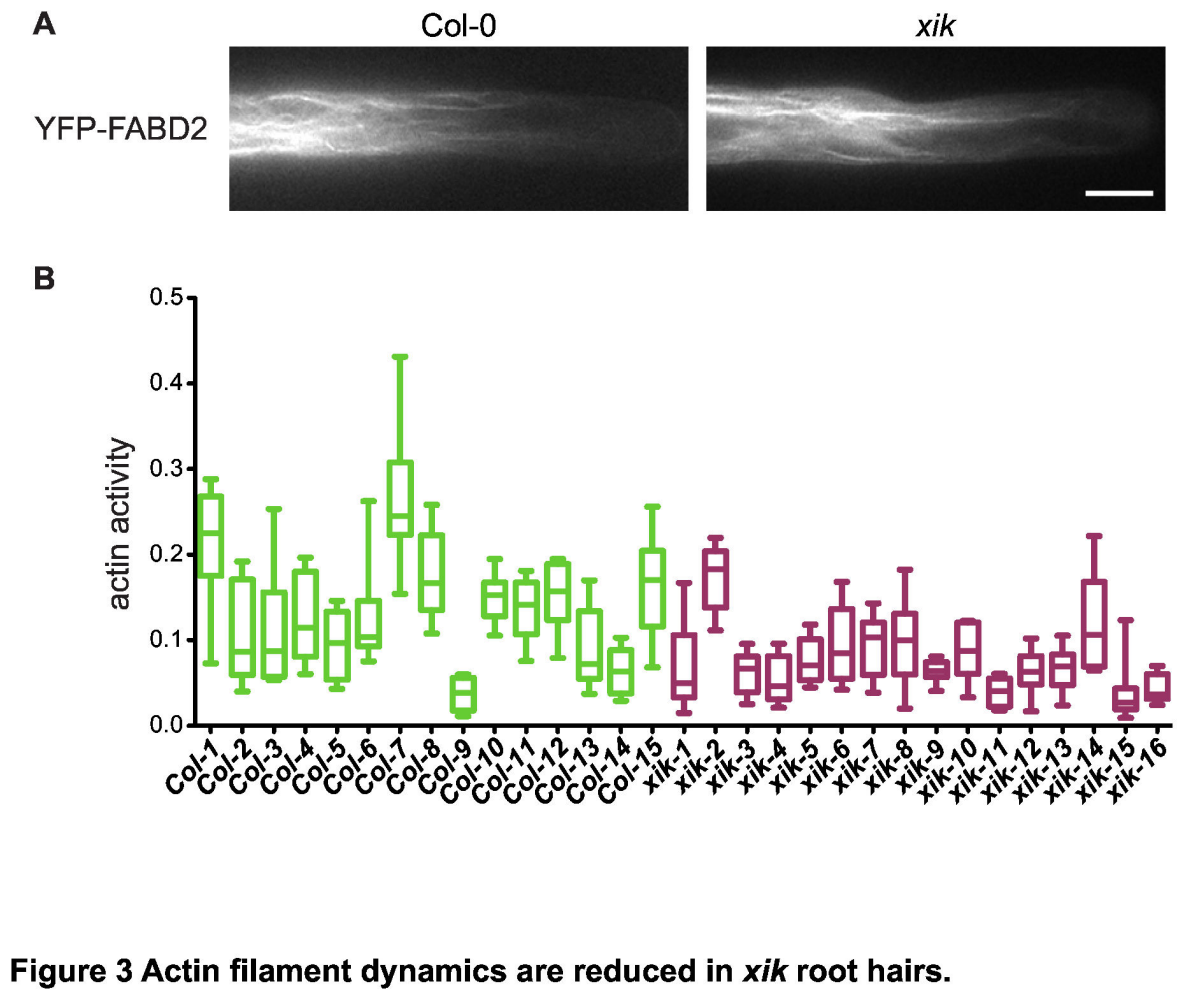
Actin filament dynamics are reduced in *xik* root hairs. (A) Actin filament organization as labeled with YFP-FABD2 is similar in *xik* and Col-0. Images are single frames from time-lapse observations in cells *Col*-3 and *xik*-3 in panel B, respectively. (B) Actin filament activity in wild type (Col) and mutant (xik) root hair cells (see Movie S2) as determined by the decay of image cross-correlation with increasing time intervals (for details see Figure S2). Box-and-whiskers plot indicate median, 25th and 75th percentile, as well as minimal and maximal decay constants for the ten most active regions in a given cell. Mean activity levels in Col-0 are significantly higher than in *xik* (Mann-Whitney test; p < 0.01).

Recent reports demonstrate that motor proteins can affect organization of the actin cytoskeleton [25,29]. To examine possible effects of *xik* in root hairs, actin organization in growing root hairs was visualized by decoration with YFP-FABD2 [46]. This marker labels the long filaments and cables along the shank of a root hair but does not label the fine actin filaments behind the growing tip [47]. Overall organization of the actin filaments was similar in *xik* and in wild type (Figure 3A), as has previously been reported for leaf petiole epidermal cells [29]. This arrangement of actin filaments, however, was not static. Time-lapse analysis revealed dramatic lateral displacements of actin cables that often appeared to travel like waves through the cell. Interestingly, these dynamic rearrangements of actin filaments appeared to be reduced in *xik* root hairs (Movie S2). Quantitative analysis of these dynamics by measuring the decay of image cross-correlation over time (see Methods and Figure S5) revealed that actin movements were significantly reduced in the mutant (Mann-Whitney test, p<0.01; Figure 3B). Thus, myosin XIK is required for normal dynamic behavior of actin filaments, but is not necessary for normal organization of actin filaments in the root hair shank.

### *XIKpro:YFP-XIK* can complement the *xik* mutant phenotype

**Figure 4:**
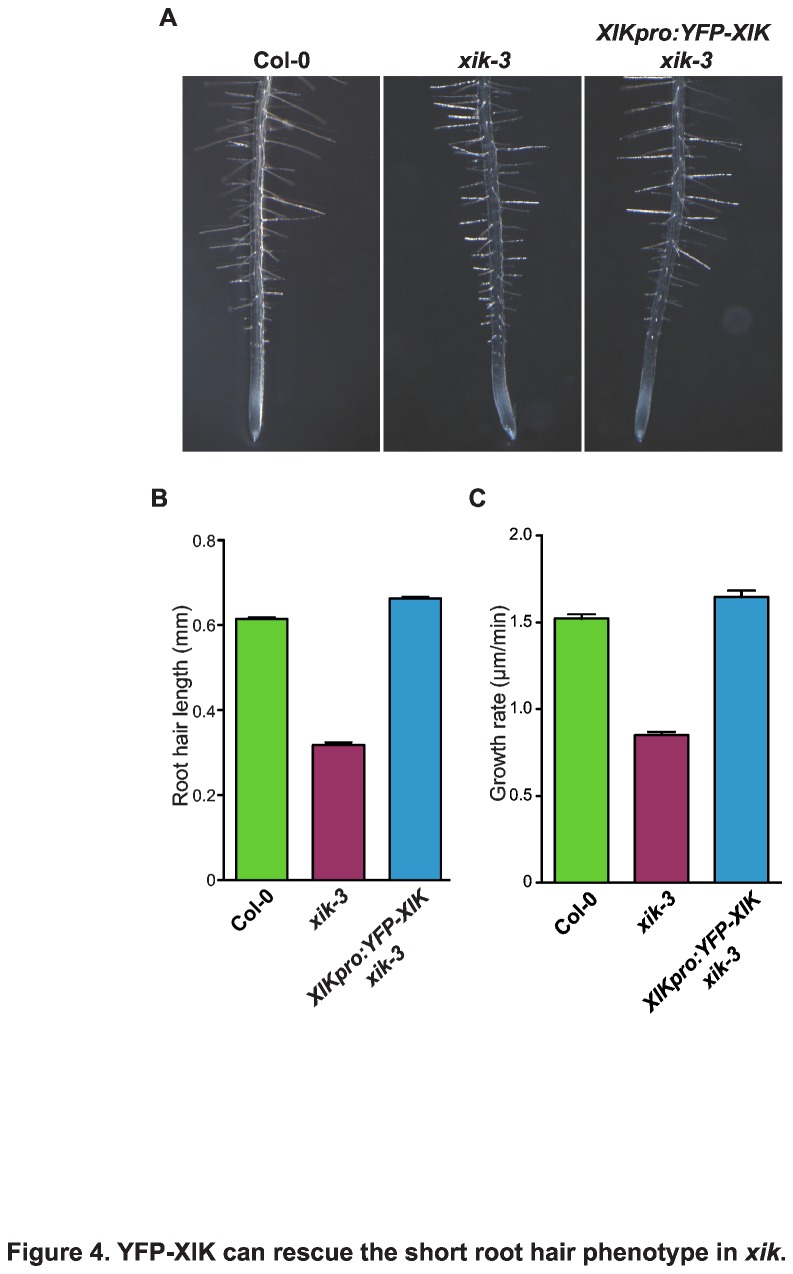
YFP-XIK can rescue the short root hair phenotype in *xik*. (A) Root hairs of *XIKpro:YFP-XIK* *xik-3* seedlings displayed long root hairs similar to those of Col-0. Pictures were taken with five day old seedlings grown on vertical growth media solidified with phytagel. (B) Lengths of mature root hairs. Root hairs of *XIKpro:YFP-XIK* *xik-3* showed similar length as those of wild type (n=730 Col-0; n=370 *xik-3*; n=660 *XIKpro:YFP-XIK* *xik-3*). Error bars represent standard error of the mean. (C) Average growth rate of root hairs in *XIKpro:YFP-XIK* *xik-3* was similar to those of Col-0. Growth rates were measured in 10 second intervals (n=1350 Col-0; n=1080 *xik-3*; n=471 *XIKpro:YFP-XIK* *xik-3*). Error bars represent standard error of the mean.

To gain a better understanding of the involvement of XIK-driven movements in root hair tip growth, it is necessary to identify the cargo of XIK. As a first step towards this goal, we wanted to determine the intracellular localization of XIK. Thus, a wild type *XIK* cDNA was cloned behind the coding region of YFP and transformed into *xik* mutants under the control of its native promoter (*XIKpro:YFP-XIK*, Figure S1A). Three individual transgenic lines were identified and their root hair length was measured. In all cases, *XIKpro:YFP-XIK* could complement *xik* root hair growth (Figure 4A). The average length of *XIKpro:YFP-XIK xik* root hairs was 0.64 ± 0.006 mm (SE, n=660, Figure 4B), which is slightly longer than that of wild type (0.61 ± 0.004 mm; n=370; t-test, p<0.0001), while *xik* produced significantly shorter root hairs (0.32 ± 0.06 mm, n=370; t-test, p<0.0001). In addition, root hair growth rates were increased back to those of wild type (Figure 4C). While the average growth rate of growing root hairs in *xik* was 0.85 ± 0.01 µm/min (SE, n= 11 root hairs; 1080 time points), *XIKpro:YFP-XIK xik* showed an average growth rate of 1.64 ± 0.02 µm/min (SE, n=16 root hairs; 471 time points), which is slightly higher than in wild type (1.52 ± 0.01 µm/min, SE, n= 13 root hairs; 1350 time points; t-test between wild type and *XIKpro:YFP-XIK xik*, p<0.05, while t-test between *xik-3* and *XIKpro:YFP-XIK xik*, p<0.0001). These results confirmed that the slower growth of root hairs in *xik* mutants resulted from the lack of XIK motor proteins. In addition, this also established that the YFP-XIK fusion protein could function normally in plants, which implies that these transgenic plants could be used to observe normal XIK localization in cells.

### YFP-XIK accumulation at the tip of growing root hairs depends on actin filaments

**Figure 5:**
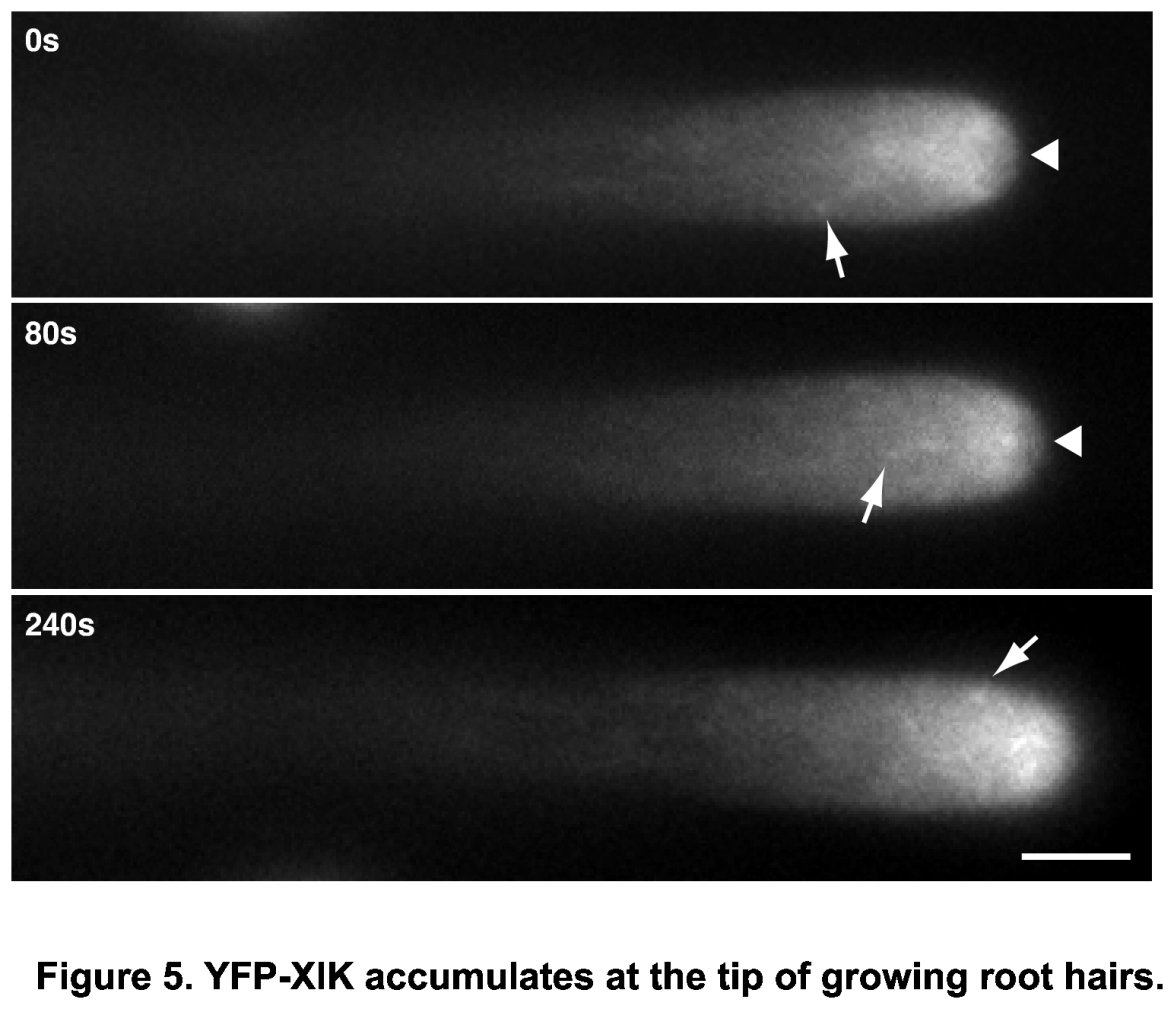
YFP-XIK accumulates at the tip of growing root hairs. Stable transgenic plants of *XIKpro:YFP-XIK* in *xik-3* background showed maximal accumulation of YFP signal in the subapex of growing root hairs. Weaker accumulation occurred in the extreme tip (arrow heads). YFP-XIK labeled vesicles are difficult to distinguish at the tip while they are easier to detect in the shank of root hairs (arrows). Images are representative frames from a 30 minute time-lapse observation (see Movie S3). Size bar indicates 10 μm.

YFP-XIK signal was low in all tissues examined, suggesting that XIK myosin protein levels are generally low in plant cells. Interestingly, relatively high accumulation of YFP was observed at the tip of growing root hairs (Figure 5). This accumulation of YFP-XIK was stable while root hairs were growing (Movie S3), but disappeared when root hairs were fully grown (data not shown and [30]). Occasionally, distinct small spots appeared in the shank of root hairs that moved both towards and away from the tip (arrows in Figure 5).

**Figure 6:**
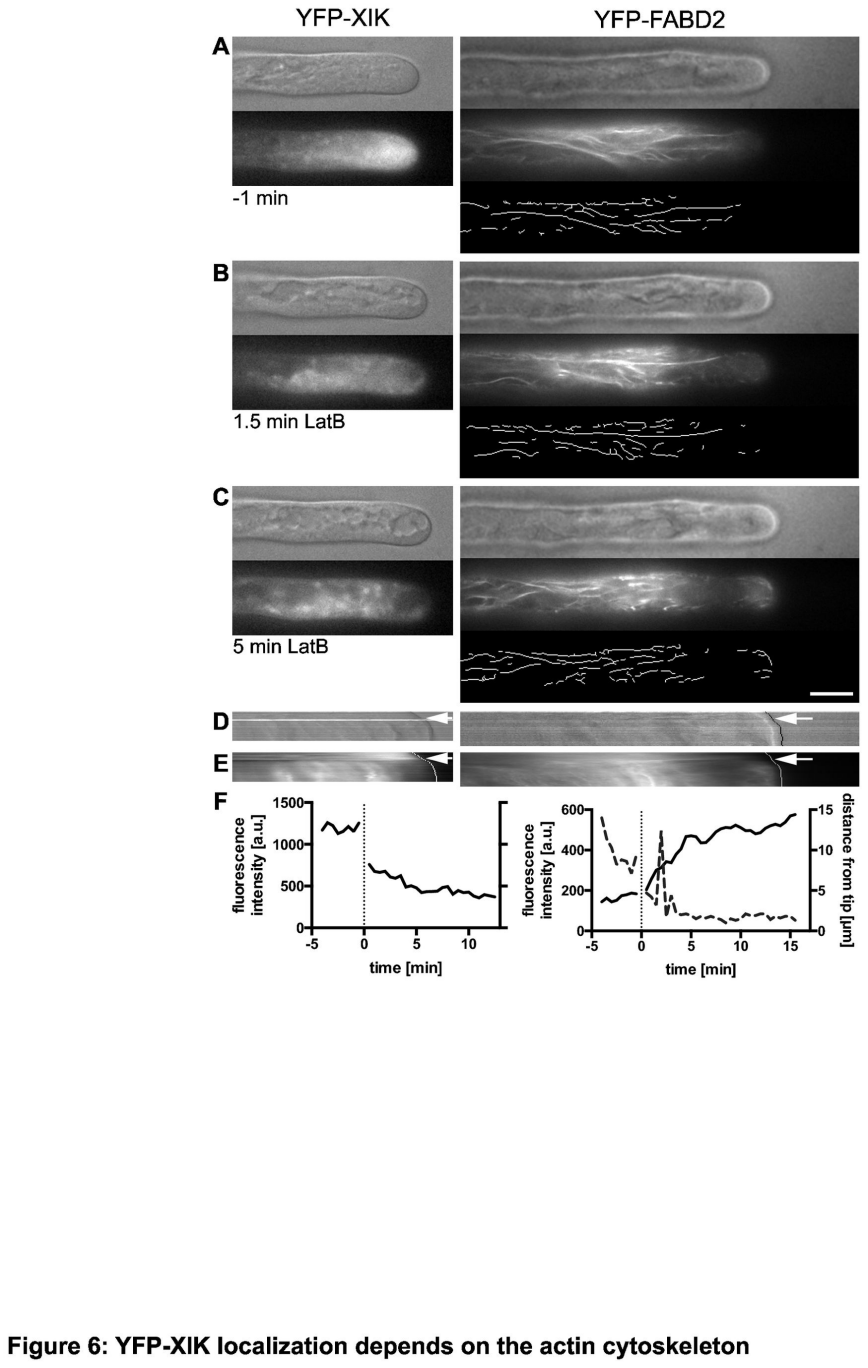
Treatment with the actin depolymerizing drug, LatB, disrupts YFP-XIK accumulation at the tip. Root hairs expressing YFP-XIK (left column) or YFP-FABD2 (right column) were treated with 100 nM latrunculin B (LatB) during observation on the microscope (n=8 for YFP-XIK; n=10 for YFP-FABD2). DIC and fluorescence images were captured in 30 s intervals. YFP-XIK images were captured near the center of the root hair, whereas YFP-FABD2 images show the cortical actin filaments. Representative images from Movies S4 and S5 at the indicated times are shown. Size bar for A-C: 10 µm. (A) Prior to treatment, YFP-XIK accumulated near the tip and actin filaments formed many, primarily longitudinal bundles in the shank. Computational detection of actin filaments is shown below the fluorescence image. Note that this actin marker does not label the fine actin mesh in the apical region of the root hair. (B) Within 1.5 min of LatB treatment, YFP-XIK intensity in the tip decreased while actin cables showed first signs of disorganization with the appearance of short filaments in the apical region. At this time, large vacuoles were seen to approach the tip (e.g. left DIC image). (C) After 5 min of LatB treatment, YFP-XIK was largely absent from the tip and formed larger clusters in the subapical and shank regions. YFP-FABD2 cables were still present in the shank and also were present in the tip region of the root hair. These areas were separated by a region with no or few actin filaments and low fluorescence intensity. (D) Kymograph of DIC images revealing growth of the root hair. Total height of the image represents 17 min (YFP-XIK) and 20 min (YFP-FABD2), respectively. Note that growth of the root hair stopped around 5 min after LatB application (arrow). (E) Kymograph of fluorescence images. YFP-XIK fluorescence was strongest near the tip before LatB application (arrow) but then dropped quickly in the tip region. By contrast, YFP-FABD2 signal in the tip was low prior to treatment but increased in the presence of LatB. Curved white lines indicate position of the root hair tip. (F) Quantitative analysis of YFP-XIK and YFP-FABD2 accumulation in the tip. Solid lines indicate fluorescence intensities of the two markers within the first 2 µm of the root hair over time. Dashed line in right graph indicates the distance of the front-most actin filament from the tip (see lower panels in A-C). Vertical dotted line indicates start of LatB treatment during which root hair shifted and focus had to be reestablished.

To test whether the tip accumulation of YFP-XIK resulted from functional XIK movement along actin filaments to deliver its cargo to the tip of a growing root hair, the actin polymerization inhibitor latrunculin B (LatB) was used to observe the effect of actin filament disruption on the localization of YFP-XIK. We employed a relatively low concentration of LatB (100 nM) that was sufficient to stop root hair growth within 5 to 7 minutes (compare Figure 6D). Consistent with previous reports [48], actin organization in the tip region of growing root hairs was rapidly altered in all 10 cells we observed (right column in Figure 6; Movie S5). Interestingly, short actin filaments appeared in the apical region that normally excluded this marker (Figure 6B, C), similar to what has been described for pollen tubes [49]. These clearly detectable filaments were accompanied by a general increase in YFP signal intensity in this area (Figure 6E, F), suggesting that additional filaments were formed that were accessible to the YFP-FABD2 marker but that could not be resolved by epifluorescence. The presence of YFP-FABD2 signal in this area of the root hair indicates that the typical fine actin mesh that normally occupies the subapical region [47] was disrupted by the LatB treatment. Long actin filaments in the shank were replaced with shorter filaments only after longer treatment times, typically after growth had already stopped (data not shown), suggesting that these filaments were relatively stable and turned over only slowly.

In parallel to the accumulation of actin in the tip region, we observed a loss of YFP-XIK signal in the same area (n=8; Figure 6, left column; Movie S4). This loss of YFP-XIK accumulation (Figure 6F) was accompanied by the appearance of vacuoles in the apical area (Figure 6B,C, DIC image), indicating a dramatic reorganization of the tip cytoplasm. During LatB treatment, YFP-XIK began to appear in several larger aggregates dispersed throughout the root hair (Figure 6C). These aggregates did not represent accumulations of cytosol since they did not coincide with the soluble mCherry marker (data not shown). Taken together, our data indicate that growing root hairs respond rapidly to moderate concentrations of LatB by reorganizing their actin cytoskeleton near the tip which is accompanied by a rapid loss of myosin XIK near the tip.

### YFP-XIK partially colocalizes with several vesicle markers that accumulate at the tip of root hairs

The accumulation of YFP-XIK in the root hair tip could result from one of several mechanisms, among them the association with RabA4b vesicles that accumulate in the apical area [8]. In addition, the Rho/Rac-like small G-protein ROP2 has been recognized as an essential regulator of tip growth that accumulates both at the apical plasma membrane and on vesicles near the apex [50]. Similarly, YFP-RHD4 has been shown to accumulate at the tip of growing root hairs and *rhd4-1* mutants showed stochastic YFP-RabA4b fluctuations [38]. Alternatively, the tip region is known to be rich in cytosol and YFP-XIK accumulation might simply reflect free diffusion into the accessible space in the tip. Thus, to gain additional insight into the mechanism of XIK accumulation at the tip and its likely cargo, we compared the distribution of YFP-XIK with several markers in root hairs of double transgenic plants.

**Figure 7:**
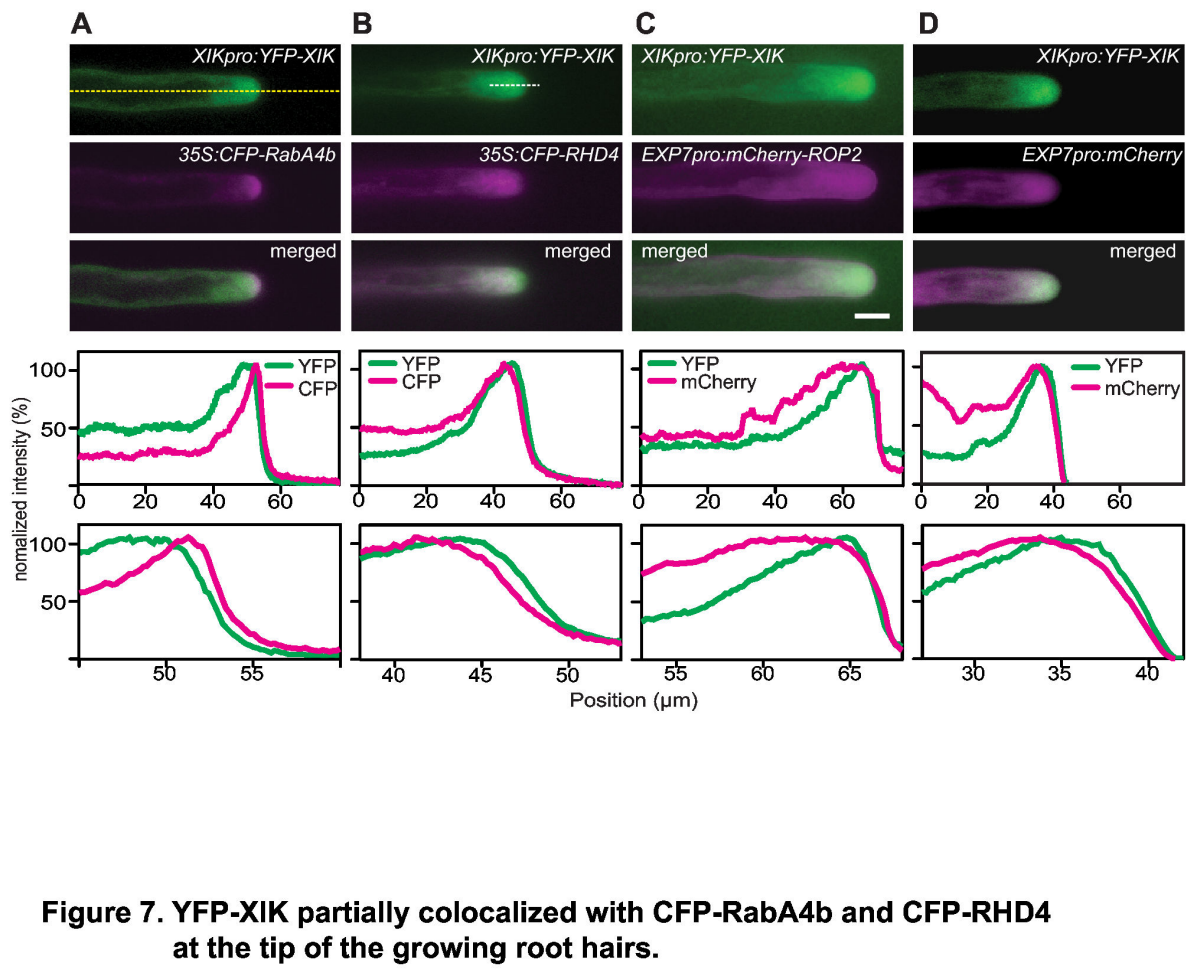
YFP-XIK partially colocalized with CFP-RabA4b and CFP-RHD4 at the tip of the growing root hairs. First row of each panel shows YFP-XIK (green) localization in the root hairs while the second row presents other markers in the same root hairs (magenta). Merged images are shown in the third row. To compare localizations, pixel intensity along the root hair center (yellow dashed line in A) of each merged image was visualized in the fourth row. Signal intensities along the first 15 μm near the tip (white dashed line in B) are shown to emphasize the distribution of the two proteins near the tip of root hairs (fifth row). Scale bar indicates 10 μm. (a) Partial colocalization of CFP-RabA4b and YFP-XIK. Although CFP-RabA4b and YFP-XIK localized at the tip of root hairs, CFP-RabA4b proteins accumulated closer to the apex while YFP-XIK signal was maximal in the subapex of the root hairs. Also compare Movie S6. (b) Partial colocalization of CFP-RHD4 and YFP-XIK. CFP-RHD4 labeled vesicles accumulated further away from the root hair tip than YFP-XIK which resulted in partial overlap with the YFP-XIK signal. (C) mCherry-ROP2 localization in cytosol and plasma membrane. Localization of YFP-XIK was clearly distinguishable from both inactive mCherry-ROP2 in the cytosol and active mCherry-ROP2 at the plasma membrane. (D) Distribution of cytosolic mCherry was different from YFP-XIK localization at the root hair tip suggesting that accumulation of YFP-XIK at the tip did not reflect free diffusion.

Interestingly, accumulation of CFP-RabA4b and YFP-XIK overlapped partially within the apical dome but the peak of CFP-RabA4b accumulation occurred closer to the apex than that of YFP-XIK (Figure 7A). Quantitative analysis of signal distribution over time revealed a stable accumulation of the CFP-RabA4b maximum approximately 3 µm ahead of YFP-XIK maximum (Table 1 and Figure S6). In addition, tip accumulation of YFP-XIK and CFP-RabA4b fluctuated independently (Movie S6), suggesting that the CFP-RabA4b labeled vesicles at the root hair apex were not associated with YFP-XIK. CFP-RHD4 was partially colocalized near the apex where YFP-XIK accumulated, however, CFP-RHD4 signal was weaker in the apical dome of the root hair tip, while YFP-XIK was highly accumulated in that area (Figure 7B). This was confirmed by a quantitative analysis of the position of maximal signal intensity in several root hair cells (Table 1 and Figure S6). A similar distribution in the cytoplasm was observed for mCherry-ROP2, except that this marker was also present at the apical plasma membrane (Figure 7C). Thus, YFP-XIK accumulation peaks closer to the apex than RHD4 or ROP2-containing vesicles. Finally, we established that YFP-XIK is not simply freely diffusing in the tip region by comparing it to a soluble cytosol marker, mCherry, and observing that their distributions in the tip did not match (Figure 7D). Specifically, we found that YFP-XIK accumulation peaked approximately 3 µm ahead of the mCherry marker (Table 1 and Figure S6).

**Table 1:** Protein distribution near the root hair tip.

	RabA4b	RHD4	Cytosol
n	7	7	9
CFP-RabA4b	2.0 ± 0.5		
YFP-XIK	5.1 ± 1.3	4.6 ± 0.5	4.1 ± 0.3
CFP-RHD4		6.8 ± 0.8	
mCherry			7.0 ± 1.0

Distance of maximal signal intensity from the tip in actively growing root hairs (growth rates ranging from 0.2 to 1.6 µm/min).

Values represent mean ± sd and are given in µm. The maxima are statistically different from each other except for the different YFP-XIK data and the CFP-RHD4 and mCherry comparison (Tukey’s Multiple Comparison Test, p>0.05).

### ROP2 recruitment to the plasma membrane is impaired in *xik* root hairs

**Figure 8:**
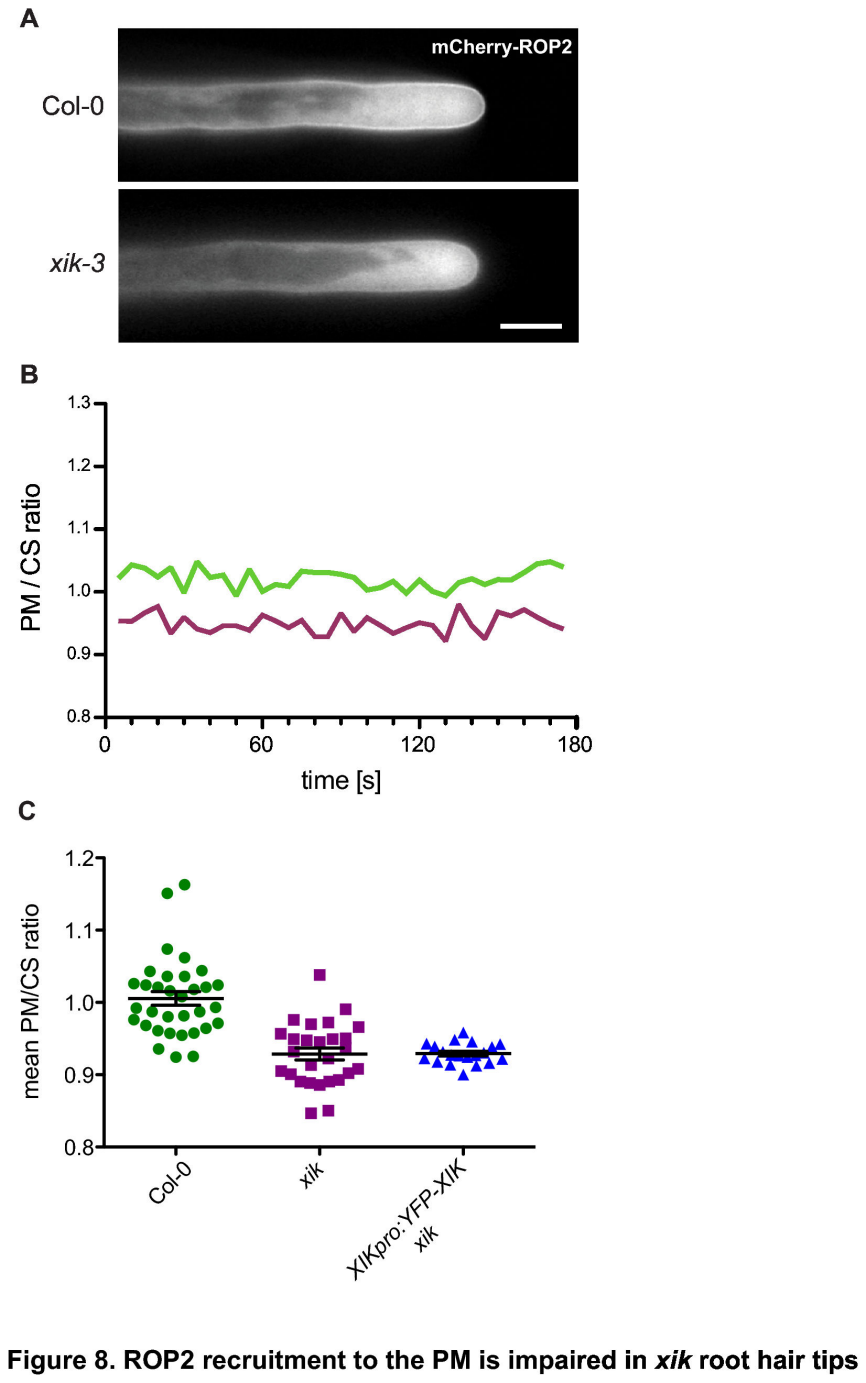
ROP2 recruitment to the plasma membrane is impaired in *xik* root hair tips. (A) Fluorescence images of mCherry-ROP2 distribution in Col-0 and *xik* root hairs. Size bar represents 15 µm. (B) Quantification of ROP2 recruitment to the plasma membrane in growing root hairs. Plasma membrane (PM) signal relative to cytosolic (CS) signal in representative root hairs of Col-0 (green) and *xik* (magenta). Measurements were taken in 5 s intervals for 3 minutes. (C) Average PM/CS ratio for root hairs from different genotypes. Every dot represents average PM/CS ratio of a single root hair over a 3 min period. Some of the Col-0 root hairs expressed YFP-ROP2 instead of mCherry-ROP2.

ROP GTPases are crucial regulators of tip growth that coordinate calcium and ROS signaling [5,51,52]. They are recruited to the plasma membrane (PM) in the tip of root hairs and pollen tubes although the inactive form of ROP proteins accumulates in the cytosol [53]. This general distribution was found in both wild type and *xik* root hairs expressing YFP-ROP2 or mCherry-ROP2 (Figure 8A), even though the signal at the PM of the mutant seemed slightly reduced. To quantify the recruitment of ROP2 to the PM, we developed an automated algorithm that determined the fluorescence signal at the tip relative to a segment of the cytosol (CS) immediately behind the apex (see Methods and Figure S3). The relative PM accumulation of ROP2 fluctuated slightly over time (Figure 8B) but the amplitude of these fluctuations was smaller (coefficient of variation about 2%) than seen for YFP-RabA4b (Figure 2C) and also did not show any periodicity as is typical for calcium concentrations [54]. Importantly, the PM recruitment was reduced in the *xik* mutant (0.93 ± 0.04, SD, n = 27) compared to wild type (1.01 ± 0.05, SD, n = 33; t-test, p<0.0001) (Figure 8C), suggesting that XIK might be involved in ROP-GTPase signaling required for tip growth but not for cell polarity.

We also tested whether expression of YFP-XIK restored normal targeting of mCherry-ROP2 to the plasma membrane. Interestingly, the PM/CS ratio of mCherry-ROP2 did not change in the complemented mutants (Figure 8C; t-test between wild type and *XIKpro:YFP-XIK xik*, p<0.0001, while t-test between *xik-3* and *XIKpro:YFP-XIK xik*, p= 0.9914), suggesting that the reduced relative abundance of the G-protein at the PM of mutant root hairs is not responsible for their slower growth. This conclusion is also supported by a lack of correlation between growth rates and PM/CS values for individual root hairs (data not shown).

## Discussion

Tip growth of root hairs presents an excellent model system to study polarized secretion in plant cells. The entire cytoplasm of hair cells is focused towards the growing tip where all secretion occurs [3]. In particular, actin filaments are arranged longitudinally along the root hair shank to facilitate movement of secretory vesicles to the growing tip where they accumulate in the apical dome (reviewed in 5). In this study, we have addressed the specific function of an actin-based molecular motor, myosin XIK, during root hair growth.

Root hairs on *xik* mutant seedlings are shorter than in wild type but appear otherwise normal. A previous study reported a slight curvature of root hairs just before cessation of growth, giving the root hairs a hook-like appearance [9]. Interestingly, a similar phenotype had been reported for the *centipede2* (*cen2*) mutant which maps to the same region of chromosome 5 as *XIK* [55] and therefore may represent a new allele of *XIK*. We have not observed this curvature under our growth conditions, suggesting that this morphological defect occurs only under some circumstances.

The normal morphology of *xik* root hairs is different from many other root hairs mutants that disrupt polar organization and lead to bulging of the tip (e.g., *tip1* [56]), wavy growth (e.g., *rhd3* [57]), or branching (e.g., *scn* [55]). This normal morphology implies that the activity of XIK is not required to maintain normal cell polarity. In other words, the complex interlocking feedback loops that occur at the tip of growing root hairs to regulate their strict polarization [5] are still in operation in *xik* mutants. This conclusion is consistent with our observation that actin filaments are arranged normally (Figure 3) and that secretory vesicles marked with YFP-RabA4b still accumulate in the apical dome in the mutant (Figure 2). Thus, the tip-focused regulatory mechanisms (termed LENS in [3]) can remain stable with the slower growth rate in *xik* and do not seem to depend on a high delivery rate of membranes to the tip. Importantly, this stability is also preserved in wild type as root hairs approach their final length and their growth rate slowly drops to zero. It should be noted, however, that simultaneous loss of several myosin genes (*mya2 xib, xi-i, xik*) can lead to defects in polarity such as branching [25] that may be masked in single mutants by the apparent redundancy of myosin paralogs.

Interestingly, we found reduced accumulation of mCherry-ROP2 at the PM in *xik* root hairs, suggesting that XIK motors are involved in regulating the partitioning of ROP2 between cytosol and the PM. This result is consistent with previous reports that overexpression of dominant negative (dn-) ROP2 resulted in premature cessation of root hair growth [58]. Thus, lack of XIK activity might result in reduced ROP2 activity and hence slower growth and earlier growth termination. This interpretation, however, is not fully supported by our data. In particular, we found that the growth rate of individual root hairs did not correlate with the PM/CS ratio in these cells (data not shown). Furthermore, expression of YFP-XIK could not restore wild type partitioning of mCherry-ROP2 (Figure 8C). These results may have been complicated by the fact that mCherry-ROP2 was vastly overexpressed from the strong *EXPANSIN7* promoter. It is possible that utilization of the native *ROP2* promoter, or of a marker that is specific for the active form ROP2, could clarify the function of XIK in regulating ROP2 membrane association.

Growth of root hairs correlates with the accumulation of RabA4b vesicles at the tip when measured at fairly long time scales of several minutes [8]. When observing root hairs at shorter time intervals, however, we were not able to detect a correlation between the relatively regular growth oscillations and the more random YFP-RabA4b fluctuations (Figure 2). This observation is in contrast to similar experiments in pollen tubes, where YFP-RabA4d accumulation was very regular and preceded increases in growth rate by 10 s [59]. It is possible that our results are complicated by the overexpression of YFP-RabA4b from the strong d35S promoter, whereas YFP-RabA4d expression in pollen was driven by the native promoter [60]. Consistent with this interpretation, we occasionally observed root hairs on the same root with vastly different YFP-RabA4b expression levels but similar growth rates (data not shown). Thus, it seems that the concentration of endogenous RabA4b vesicles is sufficient to maintain normal growth and that the presence of additional YFP-RabA4b may have masked the limits that the RabA4b availability might place on growth over short time scales.

Importantly, we observed that actin filaments, while normally arranged in root hairs of the *xik* mutant, displayed reduced dynamics (Figure 3). This result demonstrates that myosin motors not only use actin filaments as tracks along which to move their cargo, but that these motors also lead to the movement of their cytoskeletal tracks. Our results thus confirm findings derived from the application of a putative myosin inhibitor, 2,3-butanedione monoxime [48,61]. Interestingly, similar conclusions were recently reached in both budding yeast, *Saccharomyces cerevisiae* [62], and in fission yeast, *Schizosaccharomyces pombe* [63], suggesting that this interdependence of myosin motors and actin filaments is found in all eukaryotes. The data presented here do not allow us to distinguish whether this myosin effect in plant cells is direct or indirect. For example, it is possible that the myosin motors directly pull the actin cables past each other. Alternatively, myosin-driven organelle movements could result in hydrodynamic flow [64] which secondarily leads to passive movements of the actin filaments. The observation that loss of a single myosin leads to changes in actin dynamics, however, may explain the altered organization of the actin cytoskeleton after loss of several myosin genes [25,29]. More specifically, we predict that the longitudinal organization of actin filaments depends at least partially on the pulling activity of myosin motors that ultimately function to straighten out the actin cables along the long axis of the cell. At the same time, the activity of several myosins appears to be necessary to maintain the tip organization of growing root hairs as thick actin can extend into the apical dome in the *mya1 mya2 xik* triple mutant [25].

This effect of myosin XI in angiosperms is superficially similar to the role that these motors play in the moss *Physcomitrella patens*. In that case, loss of myosin XI activity leads to a loss of polarization of actin filaments in particular and the cell in general [28]. In contrast to *A. thaliana*, however, there does not seem to be a direct effect of myosin motors on actin dynamics in *P. patens* [28]. It is possible that the role of moss myosins in actin organization is more indirect, for example, by positioning actin filament nucleating proteins [65]. It remains to be seen whether angiosperm myosins function similarly, or whether they have evolved additional functions.

A crucial aspect in determining the mechanism of myosin function is to identify the cargo that is being moved by these motors. As a first step toward this goal, we have identified the intracellular localization of myosin XIK in root hair cells. We have taken advantage of a YFP-XIK fusion protein that was able to restore the growth defects of the *xik* mutant (Figure 4). The YFP fluorescence was found seemingly diffusely throughout the cell with a strong accumulation in the subapical region of growing hairs (Figure 5). This accumulation depended on actin filaments (Figure 6) and was different from several vesicle markers or freely soluble proteins (Figure 7). Interestingly, myosin of *P. patens* also accumulates at the tip of the polarly growing moss protonemata [28,65]. The YFP-XIK accumulation in root hairs, however, was more broadly distributed in the tip and not as tightly focused as myosin XIa accumulation in *P. patens* [28,65]. This difference may reflect the different organization of actin filaments in the two cell types, with a broad domain of fine actin filaments in root hairs [47] and a distinct cluster of actin filaments in protonemata [65]. We speculate that myosin XIK preferentially associates with the fine actin mesh in root hairs [47], although this has to be tested experimentally.

The broadly diffused localization of YFP-XIK in our experiments is consistent with previous reports from transient expression of truncated constructs containing only the tail domains in tobacco cells [14,16]. A similar distribution of XIK was also described recently for a XIK-YFP construct in *Arabidopsis* [30]. In this case, the authors reported the ubiquitous presence of small spots in confocal images that were interpreted as small vesicles [30]. We observed small, spot-like signals only occasionally in our wide-field epifluorescence micrographs (Figure 5), a discrepancy that could have resulted from the different imaging modalities. Importantly, both the N-terminal YFP fusion used in this study and the C-terminal fusion in [30] accumulated in the apical region of growing root hairs suggesting that this localization is necessary for tip growth. Unfortunately, it is not clear with which structures XIK associates in this part of the cell. While the pattern of XIK-YFP fluorescence is similar to that of SCAMP2-YFP [30], this has to be tested with direct colocalization experiments as similar patterns may still represent distinct distributions (Figure 7).

In conclusion, we have established that myosin XIK is required for normal movements of actin filaments. These reduced actin dynamics may explain the observed reduction in organelle movements in *xik* mutants [10] even though this motor does not seem to localize to the affected organelles (Figure 5) [30]. It is still not clear how these reduced actin dynamics lead to slower growth, but it is possible that subtle changes in ROP2 distribution or secretory vesicle accumulation could be involved. Further studies with more specific markers at lower expression levels, combined with high-resolution colocalization studies at the tip of growing root hairs should be able to shed more light on this fascinating problem.

## Supporting Information

Figure S1 ***XIK* gene structure and short root hair phenotype of xik alleles.** (A) *XIK* gene structure. Black bars indicate exons while gray bars indicate introns. Horizontal gray lines represent UTR regions and narrow black line shows genomic fragment containing promoter. Mutation sites of four identified mutants are marked in bold (Figure S1 online). *xik-3* was used throughout this study. The arrow indicates the genomic fragment of 2093 bp that was used as the *XIK* promoter. It consisted of an upstream region and ended at the beginning of the second exon of the gene. *xik-2*, -4, and -5 are alleles identified from previous studies [9,10].
(B) All isolated alleles of *xik* resulted in shorter root hairs. Two new insertion alleles in the promoter region isolated in this study (*xik-6* and *xik-7*) also produced the short root hair phenotype. However, root hairs on *xik-7* were intermediate in length between those on other mutant alleles and WT.
(EPS)

Figure S2 **Automatic tracking of YFP-RabA4b at root hair tip.** (A) Automatic tracking of growing root hair tip based on contrast. Red line indicates detection of root hair apex. Images were taken in 5 s intervals, but only every 12th image is shown (1 minute time delay). Different line widths are a scaling artifact.
(B) Tip position determined in (A) was used to place a circular selection area in the apical dome of growing root hairs. Average signal intensity in this area was used to represent YFP-RabA4b accumulation.
(EPS)

Figure S3 **Automatic tracking of YFP-ROP2 at root hair tip.** Automatic tracking of growing root hair tips based on contrast. Cyan lines indicate detection of apical plasma membrane and cytosolic sample (2 pixels = 0.4 µm behind PM). Images were taken in 5 s intervals, but only every third images is shown (15 s apart).
(EPS)

Figure S4 **Distribution of ER and phosphatidyl inositol 4-phosphate is not affected in xik root hairs.** (A) Representative images of ER-cb fluorescence distribution in growing root hairs of Col-0 (left) and *xik* (right).
(B) Distribution of YFP-PH_FAPP1_, indicating the presence of PI4P, in Col-0 (left) and *xik* (right).
(EPS)

Figure S5 **Cross correlation analysis of actin dynamics.** (A) Decay of image cross correlation with increasing time intervals for the ten most active 6 x 6 µm^2^ squares in wild-type (Col-11) and mutant (*xik*-1) root hairs. Cell names correspond to Figure 4B. Average image cross correlation over 120 time-lapse frames captured at 1 s intervals (i.e., covering 2 min) was calculated for each square at the indicated intervals. The ten "most active" 6 x 6 µm^2^ squares were selected among the overlapping set (3 µm step size between squares) based on the predicted span from a single-exponential decay curve fit (see Methods). The decay constant K from this curve fit was used as an indicator of actin activity and plotted in Figure 4B.
(B) Single fluorescence images of the two root hairs analyzed in A and "activity maps" of these root hairs. Hue depicts the value of the decay constant K according the color scale on the right. Brightness represents the span of the curve fit, i.e. the difference between the (predicted) starting value and the plateau. Squares with magenta outline were selected as "most active" and are shown in panel A as well as Figure 4B. Note that the squares shown here are only 3 x 3 µm^2^ in size while the calculation was performed on overlapping 6 x 6 µm^2^ squares.
(EPS)

Figure S6 **Distribution of fluorescent signals at the tip of growing root hair cells.** (A) Normalized signal intensity of different fluorescent markers in growing root hairs. Average signal intensities derived from seven or nine independent cells over time (see Methods). Each cell expressed two markers: CFP-RabA4b and YFP-XIK(a), CFP-RHD4 and YFP-XIK(b), mCherry and YFP-XIK(c).
(B) Average position of signal maxima for different markers in seven (RabA4b and RHD4 experiments) or 9 cells (Cherry experiment). “1:1 ratio” represents the position where normalized signals of YFP-XIK and the other marker are identical. “min/max ratio” indicates the position where the “other/YFP” ratio is either maximal (RabA4b) or minimal (RHD4, Cherry). Error bars represent sd.
(EPS)

Movie S1 **YFP-RabA4b accumulation at root hair tip.** YFP-RabA4b was imaged every 5 s in both Col-0 (top) and *xik* (bottom). Playback is 10 frames per second, i.e. 50-times accelerated.
(MOV)

Movie S2 **Actin dynamics in Col-0 and *xik*.** YFP-FABD2 was imaged in 1 s intervals in root hairs of Col-0 (upper two cells) and *xik* (lower two cells). Playback is at 10 frames per second, i.e. 10-times accelerated.
(MOV)

Movie S3 **YFP-XIK localization in growing root hairs.** YFP-XIK localization in growing root hairs. Images were taken in 10 s intervals and are played back at 20 frames per second (200-times accelerated).
(MOV)

Movie S4 **YFP-XIK response to 100 nM latrunculin B.** DIC and fluorescence time-lapse images of root hair growth and YFP-XIK localization during treatment with the actin inhibitor latrunculin B. Treatment started at time 0 min during which the root hair shifted and focus had to be re-established. Images were taken in 30 s intervals and are played back at 5 frames per second (60-times accelerated).
(MOV)

Movie S5 **YFP-FABD2 response to 100 nM latrunculin B.** DIC and fluorescence time-lapse images of root hair growth and actin filament organization during treatment with the actin inhibitor latrunculin B. Treatment started at time 0 min during which the root hair shifted and focus had to be re-established. Images were taken in 30 s intervals and are played back at 5 frames per second (60-times accelerated).
(MOV)

Movie S6 **YFP-XIK and CFP-RabA4b colocalization.** YFP-XIK (green) and CFP-XIK (magenta) were sequentially imaged. Image pairs were captured with 1 s intervals and played back at 10 fps (10-times accelerated).
(MOV)

Table S1 **Markers used in this study.**
(DOCX)
