# Supplementary material for: Myosin XIK of Arabidopsis thaliana Accumulates at the Root Hair Tip and Is Required for Fast Root Hair Growth
Source: PLoS One. 2013 Oct 7;8(10):e76745. doi: 10.1371/journal.pone.0076745 (PMC3792037; doi:10.1371/journal.pone.0076745)
Supplement: Table S1 — Markers used in this study. (DOCX) [file pone.0076745.s013.docx]

**Supplemental Tables**

**Supplemental Table S1. Markers used in this study**

| **Name** | **Localization** | **Selection marker** | **Reference** |
| --- | --- | --- | --- |
| XIKpro:YFP-XI-K | Vesicles at root hair tips | Gentamicin | (this study) |
| d35Spro:YFP-RabA4b | Vesicles at root hair tips | Hygromycin B | [Preuss et al., 2006](#_ENREF_2) |
| d35Spro:CFP-RabA4b | Vesicles at root hair tips | Hygromycin B | [Preuss et al., 2006](#_ENREF_2) |
| d35S:YFP-PH_FAPP1_ | Plasma membrane at root hair | Hygromycin B | [Vermeer et al., 2009](#_ENREF_3) |
| d35S:CFP-HDEL | Endoplasmic reticulum | Basta | [Nelson et al., 2007](#_ENREF_1) |
| d35Spro:YFP-FABD2 | Actin filaments | Basta | (this study) |
| d35Spro:CFP-RHD4 | Root hair tip accumulation | Basta | (this study) |
| EXP7pro:mCherry | Cytoplasmic localization | Basta | (this study) |
| EXP7pro:YFP-ROP2 | Plasma membrane at root hair | Basta | (this study) |
| EXP7pro: mCherry -ROP2 | Plasma membrane at root hair | Basta | (this study) |
